# Caffeic Acid Phenethyl Ester Prevents Colitis-Associated Cancer by Inhibiting NLRP3 Inflammasome

**DOI:** 10.3389/fonc.2020.00721

**Published:** 2020-05-06

**Authors:** Guoliang Dai, Zhitao Jiang, Bingting Sun, Chao Liu, Qinghai Meng, Kang Ding, Wen Jing, Wenzheng Ju

**Affiliations:** ^1^Department of Clinical Pharmacology, Affiliated Hospital of Nanjing University of Chinese Medicine, Nanjing, China; ^2^Department of Pharmacy, Zhangjiagang Hospital of Traditional Chinese Medicine Affiliated to Nanjing University of Chinese Medicine, Zhangjiagang, China; ^3^Department of Pharmacy, Nanjing Hospital of Chinese Medicine Affiliated to Nanjing University of Chinese Medicine, Nanjing, China; ^4^Department of Pharmacy, Nanjing First Hospital, Nanjing Medical University, Nanjing, China; ^5^School of Pharmacy, Nanjing University of Chinese Medicine, Nanjing, China; ^6^National Center of Colorectal Surgery, Jiangsu Integrate Colorectal Oncology Center, Nanjing Hospital of Chinese Medicine Affiliated to Nanjing University of Chinese Medicine, Nanjing, China

**Keywords:** colitis-associated cancer, NLRP3, caffeic acid phenethyl ester, IL-1β, BMDMs, THP-1 cells

## Abstract

Long-lasting inflammation in the intestinal tract renders individuals susceptible to colitis-associated cancer (CAC). The NOD-like receptor protein 3 (NLRP3) inflammasome plays a key role in the progression of inflammatory bowel disease and CAC. Therefore, identifying effective drugs that prevent CAC by targeting NLRP3 inflammasome is of great interest. Here, we aimed to evaluate the anti-inflammatory effect of caffeic acid phenethyl ester (CAPE) on bone marrow-derived macrophages (BMDMs), THP-1 cells, and azoxymethane/dextran sulfate sodium (AOM/DSS)-induced colon cancer mouse model. We also investigated the anti-tumor mechanism of CAPE. We found that CAPE decreased NLRP3 inflammasome activation in BMDMs and THP-1 cells and protected mice from colorectal cancer induced by AOM/DSS. CAPE regulated NLRP3 at the post-transcriptional level by inhibiting reactive oxygen species (ROS) production. However, CAPE did not affect NLRP3 or IL-1β transcription, but instead enhanced NLRP3 binding to ubiquitin molecules, promoting NLRP3 ubiquitination, and contributing to the anti-tumor effect in the AOM/DSS mouse model. Moreover, CAPE suppressed the interaction between NLRP3 and CSN5 but enhanced that between NLRP3 and Cullin1 both *in vivo* and *in vitro*. Altogether, our findings demonstrate that CAPE prevents CAC by post-transcriptionally inhibiting NLRP3 inflammasome. Thus, CAPE may be an effective candidate for reducing the risk of CAC in patients with inflammatory bowel disease.

## Introduction

Colorectal cancer is the third most common cancer worldwide (1). Inflammation and immunity are important determinants of tumorigenesis, affecting cancer initiation, promotion, malignant transformation, and metastasis (2). Compared with the general population, patients with inflammatory bowel disease have a much higher risk of colorectal cancer (3). In addition, patients with ulcerative colitis-associated cancer (CAC) have poorer survival than patients with sporadic colorectal cancer in the advanced stage (4). Therefore, anti-inflammatory chemopreventive intervention is of great significance to this high-risk population.

Pattern recognition receptors initiate inflammatory responses to restore homeostasis in the case of microbial or risk-related molecular patterns. Many pattern recognition receptors, including members of the NOD-like receptor family, work by assembling macromolecular complexes referred to as inflammasomes (5). In particular, the NOD-like receptor protein 3 (NLRP3) inflammasome plays crucial roles in host defense against infection, inflammation-induced cancer, and several inflammatory diseases including inflammatory bowel disease (6, 7). NLRP3 triggers innate immunity by activating caspase-1 and then cleaves immune and metabolic substrates, especially the pro-inflammatory cytokine interleukin-1β (IL-1β), which induces inflammation and promotes tumor growth (8). Thus, identifying safe and effective compounds that inhibit NLRP3 may aid CAC treatment.

Caffeic acid phenethyl ester (CAPE), a bioactive extract from propolis that can be widely found in fruits, grains and dietary supplements, is an effective antioxidant with many health benefits including anti-inflammatory, antitumor, and antimicrobial activities (9). Khan et al. (10) reported that CAPE alleviates dextran sulfate sodium (DSS)-induced colitis in mice by suppressing inflammation-triggered myeloperoxidase activity and pro-inflammatory cytokine production. However, whether CAPE can inhibit CAC and the underlying mechanism remain elusive.

In this study, we found that CAPE significantly protected mice from azoxymethane (AOM)/DSS-induced CAC by inhibiting NLRP3 inflammasome activation in macrophages. Our findings highlight a potential novel strategy for treating CAC using anti-inflammatory phytochemicals against NLRP3 inflammasome.

## Materials and Methods

### Materials

CAPE (purity >99.7%) was purchased from the National Institutes for Food and Drug Control (Beijing, China). Primary antibodies used: anti-NLRP3 (ab214185, Abcam, Cambridge, UK), anti-pro-IL-1β (16806-1-AP, Proteintech, Rosemont, IL, USA), anti-β-actin (ab8227, Abcam), anti-ASC (ab175449, Abcam), anti-ubiquitin (ab7780, Abcam), anti- Cullin1 (ab75817, Abcam), anti- CSN5 (A300-014A-M, Thermo Fisher Scientific, Waltham, MA, USA), anti-cleaved caspase-1 (#4199, Cell Signaling Technology, Danvers, MA, USA), and anti-cleaved IL-1β (#8900, Cell Signaling Technology). Secondary antibodies used include: horseradish peroxidase (HRP)-conjugated anti-rabbit IgG (ab205718, Abcam) and HRP-conjugated anti-mouse IgG (ab97023, Abcam). AOM, rotenone, ATP, and lipopolysaccharide (LPS) were purchased from Sigma-Aldrich (St. Louis, MO). DSS was obtained from MP Biomedicals LL (Solon, OH). All other reagents were purchased from Sigma-Aldrich.

### Patient Selection and Tissue Preparation

A total of 30 patients with colorectal cancer were treated at the National Center of Colorectal Surgery, Nanjing Hospital of Chinese Medicine affiliated to Nanjing University of Chinese Medicine. All patients underwent radical resection, and no patients received chemo or radiotherapy before surgery. The study was performed according to the Declaration of Helsinki and informed written consent was obtained from all patients and controls after clinicians explained the purpose, nature, and possible consequences of the study. The study protocol was approved by the Medical Ethics Committee of Nanjing Hospital of Chinese Medicine affiliated to Nanjing University of Chinese Medicine (KY2014004).

### Animals

Male C57BL/6 mice (6–8 week old) were obtained from the Model Animal Research Center of Nanjing University (Nanjing, China). The mice were housed in a specific-pathogen-free facility under controlled temperatures (22 ± 2°C) and a 12:12 h light/dark cycle. Animal welfare and experimental procedures were performed in accordance with the Guide for the Care and Use of Laboratory Animals (National Institutes of Health, Bethesda, MD) and the related ethical regulations of our university.

### Mouse Model Establishment

Mice in the drug-administered groups were fed with CAPE at corresponding doses for 2 weeks stating from AOM/DSS model establishment. After drug administration, the mice in the AOM/DSS and drug-administered groups were intraperitoneally injected with AOM at 10 mg/kg and fed a normal diet for 7 days, after which they were subjected to a repetitive DSS administration cycle (four times). In each cycle, the mice were given 2.3% DSS solution for 7 days, followed by 14 days of normal water without DSS. The control mice were fed a normal diet.

### Histomorphology

Colon tissues were fixed in 4% formaldehyde and embedded in paraffin wax. Then, 5-μm-thick sections were obtained using a microtome (Leica, Wetzlar, Germany) and placed on microscopic slides. The sections were then stained with hematoxylin and eosin as previously described (11). The severity of damage in all sections was assessed by independent pathologists.

### Western Blotting

Samples were collected, washed with ice-cold PBS, and lysed in lysis buffer. Whole sample lysates were centrifuged, after which the protein-containing supernatant was collected. Total proteins were then boiled in water, separated by sodium dodecyl sulfate-polyacrylamide gel electrophoresis (SDS-PAGE), and electrophoretically transferred onto polyvinylidene fluoride membranes (Millipore, Burlington, MA). The membranes were blocked with 5% bovine serum albumin for 1 h at room temperature, probed with primary antibodies overnight at 4°C, and then incubated with HRP-coupled secondary antibodies. Primary antibodies included NLRP3 (1:1,000), cleaved caspase-1 (1:500), pro-IL-1β (1:1,000), cleaved IL-1β (1:1,000), β-actin (1:10,000), ASC (1:1,000), and ubiquitin (1:1,000) antibodies; concentrations were determined according to manufacturers' instructions. Secondary antibodies included HRP-labeled goat anti-rabbit IgG (1:10,000) and HRP-labeled goat anti-mouse IgG (1:10,000). Blots were developed using enhanced chemiluminescence reagent (PerkinElmer, Waltham, MA). Data were analyzed with the Quantity One v-4.6.5 software (Bio-Rad Laboratories, Hercules, CA).

### Co-immunoprecipitation

Total lysates from cells or tissues were immunoprecipitated with 2 μg of appropriate antibody or the corresponding IgG control for 1 h at 4°C, and then precipitated with protein A/G-agarose beads overnight at 4°C (Santa Cruz Biotechnology, Dallas, TX). Beads were washed three times with low-salt lysis buffer for. Immunoprecipitated proteins were separated by SDS-PAGE, followed by western blotting with the corresponding antibodies.

### Cell Culture

Bone marrow-derived macrophages (BMDMs) and THP-1 cells were purchased from the American Type Culture Collection (Manassas, VA). The cells were incubated in DMEM supplemented with 10% fetal bovine serum (Life Technologies, Carlsbad, CA), 100 U/ml penicillin, and 100 mg/ml streptomycin in a humidified atmosphere at 37°C and 5% CO_2_.

### Real-Time PCR

Briefly, total RNA was extracted with Trizol reagent (Invitrogen, Carlsbad, CA) and reverse transcribed into cDNA with an real-time PCR kit (Roche, Basel, Switzerland) according to manufacturer's instructions. Real-time PCR was performed with cDNA equivalent to 200 ng of total RNA on a Bio-Rad iQ5 Real-Time PCR Detection System (Bio-Rad Laboratories). Cycling conditions were as follows, 95°C for 2 min, followed by 40 cycles of 95°C for 10 s, 60°C for 30 s, and 95°C for 10 s. GAPDH was used as an endogenous control. PCR results were normalized to GAPDH expression and were quantified by the ^ΔΔ^CT method. The sense and antisense primers used in this study are listed in Table 1. All primers were synthesized by Sangon Biotech. Co., Ltd. (Shanghai, China).

**Table 1:** Primers used for real-time PCR analysis.

		**Forward**	**Reverse**
NLRP3	H	CTAGCTGTTCCTGAGGCTGG	AGCCCTTCTGGGGAGGATAG
	M	TATCCACTGCCGAGAGGTGA	TCTTGCACACTGGTGGGTTT
IL-1β	H	CAGAAGTACCTGAGCTCGCC	AGATTCGTAGCTGGATGCCG
	M	TGCCACCTTTTGACAGTGATG	AAGGTCCACGGGAAAGACAC
TNF-α	H	CTGGGCAGGTCTACTTTGGG	CTGGAGGCCCCAGTTTGAAT
	M	AGGCACTCCCCCAAAAGATG	CCACTTGGTGGTTTGTGAGTG
IL-6	H	CTCAATATTAGAGTCTCAACCCCCA	GAGAAGGCAACTGGACCGAA
	M	CAACGATGATGCACTTGCAGA	TGTGACTCCAGCTTATCTCTTGG
GAPDH	H	AATGGGCAGCCGTTAGGAAA	GCGCCCAATACGACCAAATC
	M	CCCTTAAGAGGGATGCTGCC	ACTGTGCCGTTGAATTTGCC

*H, human; M, mouse*.

### Immunohistochemistry

Excised tumor tissues were fixed in 5% formalin for 24 h before embedding in paraffin and sectioning (5 μM thick) as previously described (11). The sections were then deparaffinized and rehydrated. After endogenous peroxidase activity was blocked with methanol and hydrogen peroxide, non-specific binding sites were blocked with 5% bovine serum albumin at 37°C for 30 min. Then, tissue sections were incubated with primary antibodies at 4°C overnight, followed by incubation with a streptavidin-peroxidase complex at 37°C for 30 min. The sections were then incubated with a secondary antibody for 60 min at room temperature. The positively stained sites were visualized by incubating with peroxidase-labeled streptavidin-complexed DAB, and nuclei were counterstained with hematoxylin. Yellowish-brown staining indicates immune-positive sites and blue or purple staining indicates nuclei. Images were captured with an Olympus BX51 microscope (Tokyo, Japan).

### Intracellular Reactive Oxygen Species (ROS) Determination

Cells (1 × 10^6^ per well) were cultured in 6-well plates and treated with CAPE in the presence or absence of LPS or ATP + LPS for 6 h. Afterwards, the cells were harvested, incubated with 2′,7′-dichlorofluorescein diacetate (Invitrogen) at 37°C for 20 min, and washed twice with cold PBS. The fluorescence distribution of 2′,7′-dichlorofluorescein was detected by flow cytometry on an Accuri® C6 fluorescence-activated cell sorter (Becton Dickinson, Franklin Lakes, NJ) at an excitation wavelength of 488 nm and an emission wavelength of 525 nm.

### Enzyme-Linked Immunosorbent Assay (ELISA)

Cells were primed with LPS for 6 h (10 ng/ml), after which the medium was replaced with serum-free medium containing DMSO (1:1,000) for 1 h, followed by incubation with ATP for 1 h (5 nM). The concentrations of IL-1β, IL-6, and TNF-α in the culture supernatant were analyzed using commercially available IL-1β, IL-6, and TNF-α ELISA kits (Boster Bio, Pleasanton, CA) according to manufacturer's instructions.

### Statistical Analysis

Statistical analysis was carried out using two-tailed Student's *t*-test for comparisons between two groups and one-way analysis of variance followed by Dunnett's test for comparisons between three or more groups. Results were expressed as mean ± standard deviation (SD). *P* < 0.05 was considered statistically significant. All analyses were performed with GraphPad Prism v5.01 (GraphPad Software Inc., La Jolla, CA).

## Results

### CAPE Decreases NLRP3 Inflammasome Activation in BMDMs and THP-1 Cells

**Figure 1:**
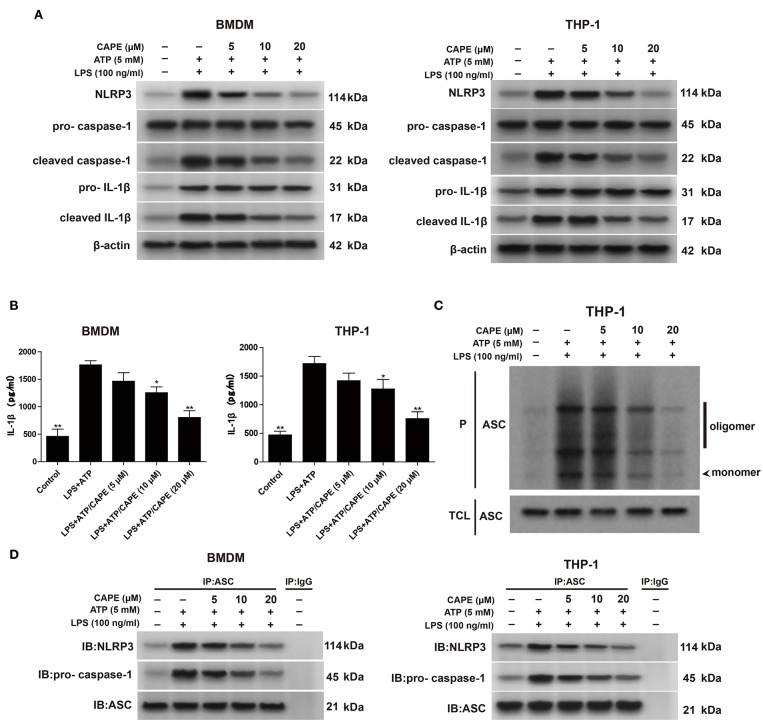
CAPE decreases the activation of NLRP3 inflammasome in BMDMs and THP-1 cells. **(A)** Effect of CAPE on the expression of NLRP3, cleaved caspase-1, pro-caspase-1, cleaved IL-1β and pro-IL-1β in BMDMs and THP-1 cells. **(B)** ELISA for IL-1β in supernatants from the indicated groups. **(C)** ASC polymerisation (oligomerization) was analyzed by Western blotting. **(D)** Coimmunoprecipitation assay using cell lysates from the indicated groups for analyzing the interaction between ASC and NLRP3 or between ASC and pro-caspase-1. Data are presented as mean ± SD (*n* = 3). *P < 0.05, **P < 0.01 vs. LPS+ATP group.

We first investigated whether CAPE inhibits the activation of NLRP3 inflammasome induced by ATP and LPS in macrophages *in vitro*. Western blotting showed that CAPE significantly inhibited the increased protein levels of NLRP3, caspase-1, and IL-1β in BMDMs and THP-1 cells after LPS and ATP stimulation (Figure 1A). Similarly, ELISA showed that CAPE significantly suppressed the secretion of IL-1β induced by LPS and ATP (Figure 1B). Moreover, CAPE significantly inhibited the formation of ASC dimers and reduced the abundance of NLRP3 inflammasome complexes in a dose-dependent manner (Figures 1C,D). Altogether, these results indicate that CAPE reduces NLRP3 protein levels and suppresses NLRP3 activation in macrophages.

### CAPE Does Not Affect NLRP3 mRNA Levels

**Figure 2:**
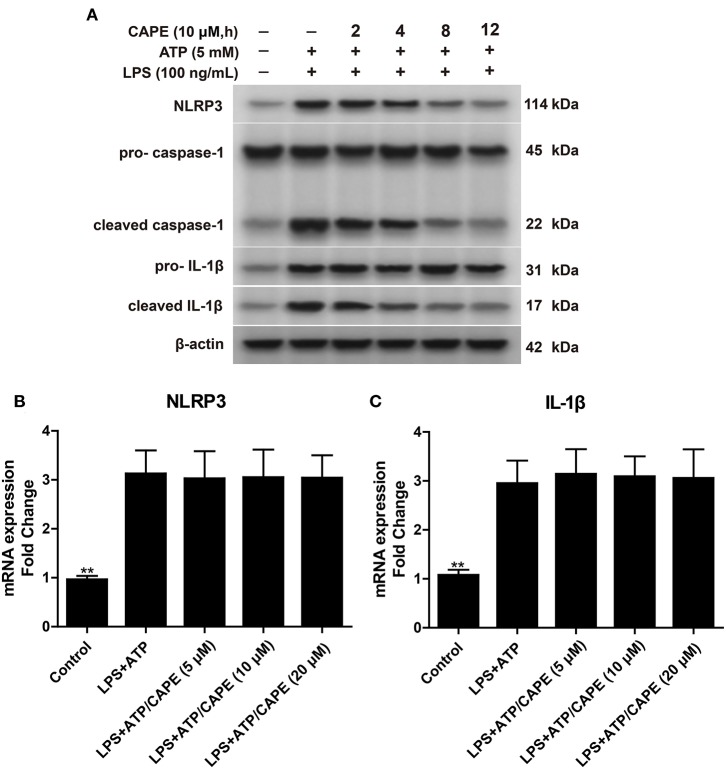
CAPE does not affect mRNA levels of NLRP3, despite altering its protein levels. **(A)** Effect of CAPE on the expression of NLRP3, cleaved caspase-1, pro-caspase-1, cleaved IL-1β, and pro-IL-1β in THP-1 cells. **(B,C)** mRNA levels of NLRP3 and IL-1β were detected by real-time PCR after CAPE treatment for 12 h. Data are presented as mean ± SD (*n* = 3). ^**^P < 0.01 vs. LPS+ATP group.

We then examined whether CAPE also reduces NLRP3 mRNA levels. As shown in Figure 2A, LPS + ATP promoted the expression of NLRP3 and pro-IL-1β in THP-1 cells; however, real-time PCR revealed that after treatment with CAPE for 12 h, mRNA levels of NLRP3 and IL-1β in THP-1 cells were similar to control (Figures 2B,C), indicating that CAPE does not affect the transcription of NLRP3 and IL-1β.

### CAPE Promotes NLRP3 Ubiquitination by Inhibiting ROS

**Figure 3:**
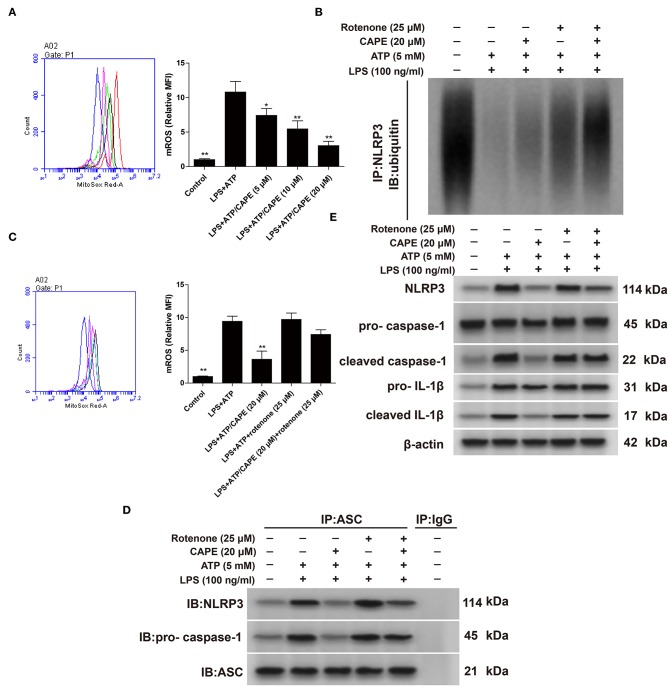
CAPE promotes NLRP3 ubiquitination by inhibiting ROS in THP-1 cells. **(A,C)** Effect of CAPE on mitochondrial production of ROS. **(B)** Cell extracts from indicated groups were subjected to immunoprecipitation assays with an anti-NLRP3 antibody, followed by Western blotting with an anti-ubiquitin antibody. **(D)** Cell lysates were subjected to immunoprecipitation assays with an anti-ASC antibody, using mouse IgG as control. **(E)** Effect of CAPE on the expression of NLRP3, cleaved caspase-1, pro-caspase-1, cleaved IL-1β, and pro-IL-1β in THP-1 cells. Data are presented as mean ± SD (*n* = 3). ^*^P < 0.05, ^**^P < 0.01 vs. LPS + ATP group.

ROS are central to the regulation of NLRP3 activation (12). Therefore, we evaluated the impact of CAPE on ROS. As shown in Figures 3A,C, CAPE significantly inhibited the production of ROS induced by LPS + ATP in THP-1 cells in a dose-dependent manner, which was reversed by rotenone. Moreover, CAPE enhanced the binding of NLRP3 to ubiquitin molecules, promoted NLRP3 ubiquitination (Figure 3B), and significantly blocked the formation of NLRP3 inflammasome, which were again reversed by rotenone (Figure 3D). Furthermore, CAPE significantly reduced the expression of NLRP3, cleaved caspase-1, and cleaved IL-1β, which was restored by rotenone (Figure 3E). Taken together, the findings indicate that CAPE regulates the expression of NLRP3 at the post-transcriptional level by inhibiting ROS production.

### CAPE Protects Mice From Colorectal Cancer Induced by AOM/DSS

**Figure 4:**
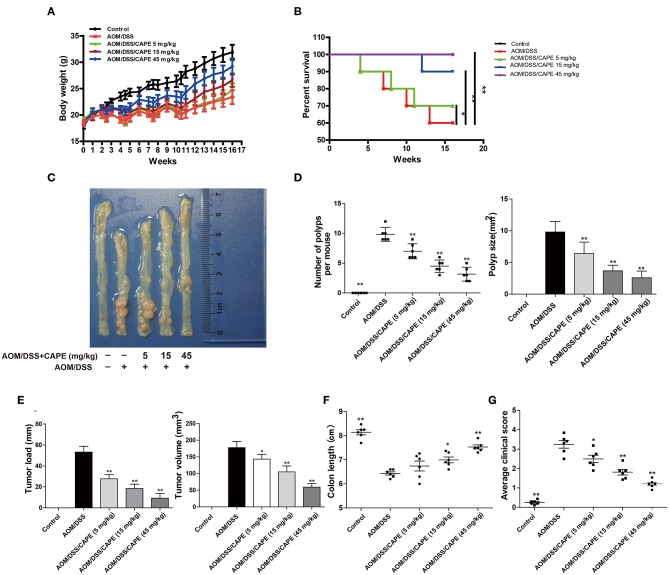
CAPE alleviates mouse colitis progression and tumor burden resulting from AOM/DSS treatment. **(A**–**G)** Effect of CAPE on **(A)** body weight, **(B)** survival rate, **(C)** intestinal tract (representative image), **(D)** number of polyps and polyp size, **(E)** tumor load and tumor size, **(F)** colon length and **(G)** average clinical score. Data are presented as mean ± SD (*n* = 3). *P < 0.05, **P < 0.01 vs. AOM/DSS group.

Subsequently, we examined whether CAPE had therapeutic effects on AOM/DSS-treated mice. The AOM/DSS group exhibited significant body weight reduction compared with that of the control group; this loss in body weight was attenuated by CAPE in a dose-dependent manner (Figure 4A). The survival rates of CAPE-administered groups were also significantly higher than those of the AOM/DSS group, and no mouse died when administered a high-dose of CAPE (45 mg/kg; Figure 4B). Moreover, CAPE administration significantly mitigated colitis progression and tumor burden. As shown in Figures 4C–F, the number, size, burden, and volume of tumors in CAPE-administered groups were significantly lower than those of the AOM/DSS group, and CAPE significantly relieved intestinal atrophy and increased colon length in a dose-dependent manner. In addition, the clinical scores of CAPE-administered groups were significantly lower than those of the AOM/DSS group, with a high-dose of CAPE (45 mg/kg) exhibiting the best efficacy (Figure 4G). Altogether, the findings demonstrate that CAPE alleviates mouse colitis progression and tumor burden caused by AOM/DSS.

### Inhibition of NLRP3 Inflammasome Contributes to the Anti-tumor Effect of CAPE

**Figure 5:**
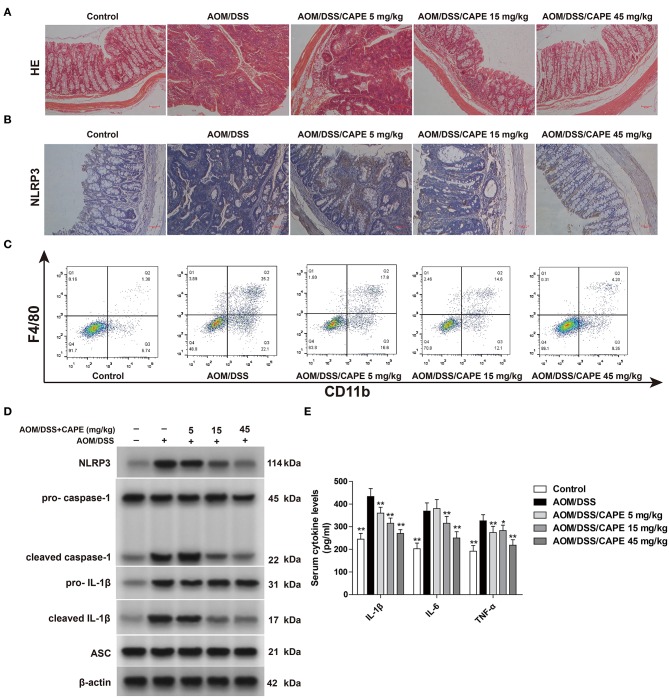
NLRP3 inflammasome inhibition contributes to the anti-tumor effect of CAPE. **(A)** Colon tissues were fixed and stained with hematoxylin-eosin. **(B)** Immunohistochemistry of NLRP3 in the colon. **(C)** F4/80^+^ CD11b^+^ macrophages in infiltrated leukocytes of colon tissue were detected by FACS blots. **(D)** Western blotting for NLRP3, pro-caspase-1, cleaved caspase-1, pro-IL-1β, cleaved IL-1β, and ASC in colons. **(E)** ELISA for IL-1β, IL-6, and TNF-α in serum. Data are represented as mean ± SD (*n* = 3). ^*^P < 0.05, ^**^P < 0.01 vs. AOM/DSS group. Scale bar = 50 μm.

To determine whether CAPE inhibits NLRP3 inflammasome *in vivo*, we assessed NLRP3 expression in the AOM/DSS mouse model by immunohistochemistry and western blotting. Hematoxylin-eosin staining demonstrated severe inflammation in the intestinal tract, and the inflammatory response and tumor formation resulting from AOM/DSS was attenuated by CAPE (Figure 5A). Moreover, CAPE significantly reduced NLRP3 accumulation in the intestinal tract (Figure 5B) and significantly inhibited AOM/DSS-induced recruitment of macrophages to intestinal tissues (Figure 5C). CAPE also significantly reduced AOM/DSS-induced expression of NLRP3, cleaved caspase-1, cleaved IL-1β, and ASC in the intestinal tract (Figure 5D). The increased secretion of IL-1β, IL-6, and TNF-α in serum was similarly reduced by CAPE in a dose-dependent manner (Figure 5E). Overall, the results indicate that activated NLRP3 in AOM/DSS mouse model is suppressed by CAPE.

### CAPE Increases NLRP3 Ubiquitination in AOM/DSS Mouse Model

**Figure 6:**
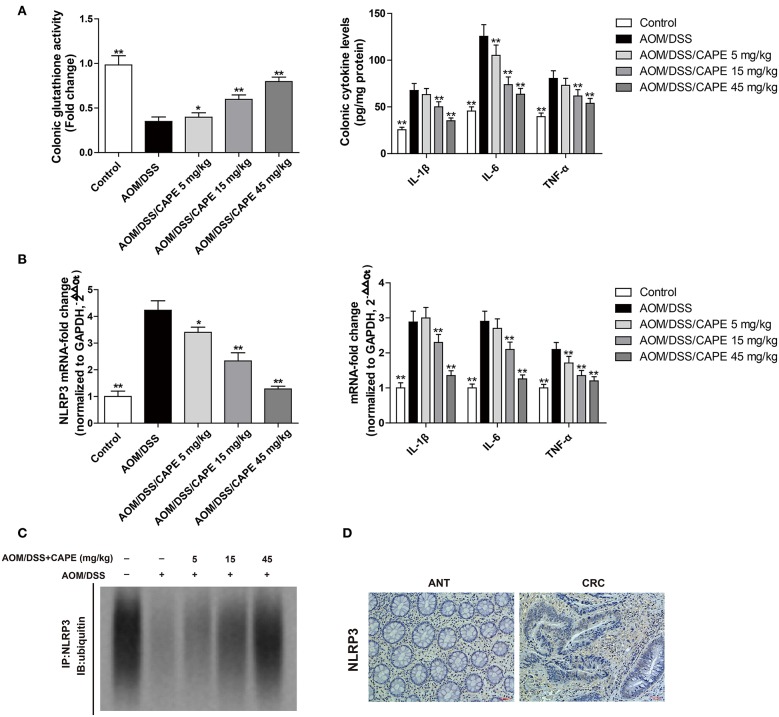
CAPE increases NLRP3 ubiquitination in the AOM/DSS mouse model. **(A)** Effect of CAPE on the activity of glutathione and the production of IL-1β, IL-6, and TNF-α. **(B)** Real-time PCR for determining mRNA levels of NLRP3, IL-1β, IL-6, and TNF-α. **(C)** Tissue extracts were subjected to immunoprecipitation with an anti-NLRP3 antibody, followed by immunoblotting with an anti-ubiquitin antibody. **(D)** Immunohistochemistry of NLRP3 expression in tumor and adjacent tissue of patient with colorectal cancer. Data are presented as mean ± SD (*n* = 3). ^*^P < 0.05, ^**^P < 0.01 vs. AOM/DSS group. Scale bar = 50 μm.

Glutathione is one of the main endogenous antioxidants produced by cells and is directly involved in the neutralization of ROS (13). The content of reduced glutathione in the intestinal tract of the AOM/DSS group was decreased; however, CAPE dose-dependently enhanced glutathione activity and reduced IL-1β, IL-6, and TNF-α levels (Figure 6A). Moreover, CAPE decreased the mRNA levels of NLRP3, IL-1β, IL-6, and TNF-α (Figure 6B), increased the binding of NLRP3 to ubiquitin molecules and facilitated NLRP3 ubiquitination (Figure 6C). In patients with colorectal cancer, NLRP3 was found highly expressed (Figure 6D). These findings indicate that CAPE also enhances NLRP3 ubiquitination *in vivo*.

### CAPE Suppresses Interaction Between NLRP3 and CSN5, and Enhances the Interaction Between NLRP3 and Cullin1

**Figure 7:**
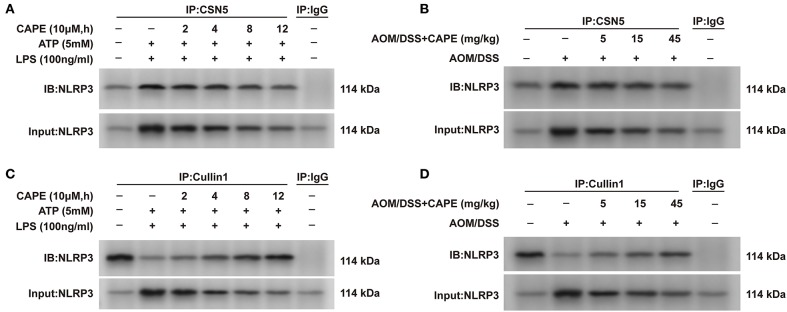
CAPE suppresses the interaction between NLRP3 and CSN5, but enhances the interaction between NLRP3 and Cullin1. **(A,C)** Immunoprecipitation assay using cell lysates with anti-CSN5 or anti-Cullin1 antibodies; mouse IgG was used as control. **(B,D)** Colon tissues lysates were subjected to immunoprecipitation assays with anti-CSN5 or anti-Cullin1 antibodies; mouse IgG was used as control. (*n* = 3).

Considering that ubiquitination is mediated by a balance between ubiquitin-conjugating and deubiquitinating enzymes (14), we examined the interaction between NLRP3 and the ubiquitin-conjugating enzyme Cullin1 and deubiquitinating enzyme CSN5. CAPE enhanced the interaction between NLRP3 and Cullin1 and decreased the interaction between NLRP3 and CSN5 in THP-1 cells in a time-dependent manner (Figures 7A,C). These findings were also observed in the AOM/DSS mouse model and occurred in a dose-dependent manner (Figures 7B,D). Thus, CAPE suppresses the interaction between NLRP3 and deubiquitinating enzymes, and enhances its interaction with a ubiquitin-conjugating enzyme *in vivo* and *in vitro*, promoting NLRP3 ubiquitination.

## Discussion

In this study, NLRP3 was found highly expressed in the tumor tissues of patients with colorectal cancer. Administering CAPE suppressed NLRP3 protein expression *in vitro* and *in vivo* by inhibiting ROS and increasing NLRP3 ubiquitination. Subsequently, inhibiting NLRP3 inflammasome by CAPE protected mice from AOM/DSS-induced CAC. Altogether, our findings indicate that inhibition of NLRP3 inflammasome by CAPE prevents CAC.

NLRP3 interacts with ASC and pro-caspase-1 to form an inflammasome. Activated NLRP3 promotes pro-caspase-1 proteolysis into its active form, caspase-1 (p20), and then cleaves pro-IL-1β and pro-IL-18 into their mature forms (IL-1β and IL-18). Macrophage-derived IL-1β stimulates Wnt signaling and leads to proliferation of colon cancer cells; high IL-1β secretion is associated with malignant phenotypes in the cancer microenvironment (15, 16). Here, CAPE inhibited IL-1β both *in vitro* and *in vivo*, highlighting its potential to suppress inflammation and CAC.

Chronic inflammation is an important event in carcinogenesis and tumor progression, and cancer-associated inflammation has been identified as the seventh hallmark of cancer (17). NLRP3 plays a key role in inflammation, and activation of NLRP3 inflammasome has been linked to inflammation-induced cancer (6, 18). Indeed, NLRP3 is activated in patients with inflammatory bowel disease and overexpression and constitutive activation of NLRP3 inflammasome contribute to the progression of head and neck squamous cell carcinoma (19, 20), lung cancer, and colorectal cancer (21, 22). Moreover, NLRP3 inhibition was found to prevent CAC (23). In agreement with these findings, we found that NLRP3 is highly expressed in the tumor tissues of patients with colorectal cancer and that CAPE protected mice from AOM/DSS-induced CAC by inhibiting NLRP3 inflammasome.

Nevertheless, the role of NLRP3 in tumorigenesis is complex. Wei et al. found that the expression of all NLRP3 inflammasome components was either completely lost or significantly downregulated in human hepatocellular carcinoma and was correlated with advanced stage and poor pathological differentiation (24). Allen et al. also reported that Nlrp3^−/−^ mice presented with acute and recurring colitis and CAC, suggesting that NLRP3 functions as a negative regulator of tumorigenesis during CAC (25). Thus, NLRP3 expression levels may vary between different tumors or stages of tumor development and NLRP3 may exert various effects via different mechanisms.

The production of ROS has been associated with cancer promotion and is implicated in the regulation of NLRP3 inflammasome (12, 26, 27). Bauernfeind et al. previously demonstrated that mitochondrial ROS regulates NLRP3 by blocking the priming step of NLRP3 inflammasome activation (28). Juliana et al. also found that mitochondrial ROS are required for the non-transcriptional priming of NLRP3 and that NLRP3 deubiquitination is a prerequisite for its activation (29). Here, CAPE did not affect the mRNA level of NLRP3, but decreased its protein levels by facilitating ubiquitination, which was abolished by rotenone, suggesting that CAPE exhibits inhibitory effects by suppressing mitochondrial ROS production.

Indeed, CAPE possessed antioxidant activity and has been reported to scavenge ROS (9, 30, 31). The anti-inflammatory effect of CAPE can most likely be attributed to suppression of ROS production at the transcriptional level by inhibiting NF-κB activation (32). Khan et al. reported that CAPE can suppress inflammation-induced MPO activity and pro-inflammatory cytokine production and can enhance epithelial barrier function in experimental colitis (10). Some recent studies also demonstrated that CAPE has a protective effect on colitis (33, 34). However, it remains unknown whether CAPE can inhibit CAC and what the underlying mechanism is. In this study, we provide evidence that CAPE facilitates NLRP3 ubiquitination by inhibiting ROS in THP-1 cells and inhibits enteritis and tumor burden by inhibiting NLRP3 in an AOM/DSS mouse model. All flavonoids in propolis, except for CAPE, have low acute oral toxicity, with an LD_50_ of 8–40 g/kg (35). In our study, the CAPE dosage at which the anti-CAC effect was evident was 45 mg/kg indicating that such a high dose of these agents is safe for preventing CAC. Further studies are needed to verify the efficiency and safety of CAPE before use in clinic.

In conclusion, CAC can be prevented by CAPE-induced NLRP3 inflammasome inhibition, highlighting CAPE as a potential candidate for reducing the risk of CAC in patients with inflammatory bowel disease.

## Data Availability Statement

All datasets generated for this study are included in the article/supplementary material.

## Ethics Statement

The studies involving human participants were reviewed and approved by Medical Ethics Committee of Nanjing Hospital of Chinese Medicine affiliated to Nanjing University of Chinese Medicine. The patients/participants provided their written informed consent to participate in this study. The animal study was reviewed and approved by Ethics Committee of Nanjing University of Chinese Medicine.

## Author Contributions

GLD participated in study design, performed statistical analysis, and drafted the manuscript. ZTJ carried out histomorphological analysis, and western blot analysis. BTS carried out cell culture and *in vitro* cytotoxicity assay. CL and WJ performed the animal experiments. QHM and KD performed real-time PCR and immunohistochemistry. WZJ conceived of the study and participated in its design and coordination. All authors read and approved the final manuscript.

## Conflict of Interest

The authors declare that the research was conducted in the absence of any commercial or financial relationships that could be construed as a potential conflict of interest.

## References

[1] Ferlay J Soerjomataram I Dikshit R Eser S Mathers C Rebelo M. Cancer incidence and mortality worldwide: sources, methods and major patterns in GLOBOCAN 2012. Int J Cancer. (2015) 136:359–86. 10.1002/ijc.29210 25220842

[2] Grivennikov SI Greten FR Karin M. Immunity, inflammation, and cancer. Cell. (2010) 140:883–99. 10.1016/j.cell.2010.01.025 20303878 PMC2866629

[3] Kim ER Chang DK. Colorectal cancer in inflammatory bowel disease: the risk, pathogenesis, prevention and diagnosis. World J Gastroenterol. (2014) 20:9872–81. 10.3748/wjg.v20.i29.9872 25110418 PMC4123369

[4] Watanabe T Konishi T Kishimoto J Kotake K Muto T Sugihara K. Ulcerative colitis-associated colorectal cancer shows a poorer survival than sporadic colorectal cancer: a nationwide Japanese study. Inflamm Bowel Dis. (2011) 17:802–8. 10.1002/ibd.21365 20848547

[5] Latz E Xiao TS Stutz A. Activation and regulation of the inflammasomes. Nat Rev Immunol. (2013) 13:397–411. 10.1038/nri3452 23702978 PMC3807999

[6] Lin C Zhang J. Inflammasomes in Inflammation-Induced Cancer. Front Immunol. (2017) 8:271. 10.3389/fimmu.2017.00271 28360909 PMC5350111

[7] Ozaki E Campbell M Doyle SL. Targeting the NLRP3 inflammasome in chronic inflammatory diseases: current perspectives. J Inflamm Res. (2015) 8:15–27. 10.2147/JIR.S51250 25653548 PMC4303395

[8] Dupaul-Chicoine J Arabzadeh A Dagenais M Douglas T Champagne C Morizot A. The Nlrp3 inflammasome suppresses colorectal cancer metastatic growth in the liver by promoting natural killer cell tumoricidal activity. Immunity. (2015) 43:751–63. 10.1016/j.immuni.2015.08.013 26384545

[9] Murtaza G Karim S Akram MR Khan SA Azhar S Mumtaz A. Caffeic acid phenethyl ester and therapeutic potentials. Biomed Res Int. (2014) 2014:145342. 10.1155/2014/145342 24971312 PMC4058104

[10] Khan MN Lane ME McCarron PA Tambuwala MM. Caffeic acid phenethyl ester is protective in experimental ulcerative colitis via reduction in levels of pro-inflammatory mediators and enhancement of epithelial barrier function. Inflammopharmacology. (2018) 26:561–9. 10.1007/s10787-017-0364-x 28528363 PMC5859149

[11] Fischer AH Jacobson KA Rose J Zeller R. Hematoxylin and eosin staining of tissue and cell sections. CSH Protoc. (2008) 2008:pdb.prot4986. 10.1101/pdb.prot4986 21356829

[12] Tschopp J Schroder K. NLRP3 inflammasome activation: the convergence of multiple signalling pathways on ROS production? Nat Rev Immunol. (2010) 10:210–5. 10.1038/nri2725 20168318

[13] Mailloux RJ McBride SL Harper ME. Unearthing the secrets of mitochondrial ROS and glutathione in bioenergetics. Trends Biochem Sci. (2013) 38:592–602. 10.1016/j.tibs.2013.09.001 24120033

[14] Liu J Shaik S Dai XP Wu Q Zhou XX Wang ZW. Targeting the ubiquitin pathway for cancer treatment. Biochim Biophys Acta. (2015) 1855:50–60. 10.1016/j.bbcan.2014.11.005 25481052 PMC4312704

[15] Kaler P Augenlicht L Klampfer L. Macrophage-derived IL-1beta stimulates Wnt signaling and growth of colon cancer cells: a crosstalk interrupted by vitamin D3. Oncogene. (2009) 28:3892–902. 10.1038/onc.2009.247 19701245 PMC2783659

[16] Zitvogel L Kepp O Galluzzi L Kroemer G. Inflammasomes in carcinogenesis and anticancer immune responses. Nat. Immunol. (2012) 13:343–51. 10.1038/ni.2224 22430787

[17] Colotta F Allavena P Sica A Garlanda C Mantovani A. Cancer-related inflammation, the seventh hallmark of cancer: links to genetic instability. Carcinogenesis. (2009) 30:1073–81. 10.1093/carcin/bgp127 19468060

[18] Davis BK Wen H Ting JP. The inflammasome NLRs in immunity, inflammation, and associated diseases. Annu Rev Immunol. (2011) 29:707–35. 10.1146/annurev-immunol-031210-101405 21219188 PMC4067317

[19] Lazaridis LD Pistiki A Giamarellos-Bourboulis EJ Georgitsi M Damoraki G Polymeros D. Activation of NLRP3 inflammasome in inflammatory bowel disease: differences between crohn's disease and ulcerative colitis. Dig Dis Sci. (2017) 62:2348–56. 10.1007/s10620-017-4609-8 28523573

[20] Huang CF Chen L Li YC Wu L Yu GT Zhang WF. NLRP3 inflammasome activation promotes inflammation-induced carcinogenesis in head and neck squamous cell carcinoma. J Exp Clin Cancer Res. (2017) 36:116. 10.1186/s13046-017-0589-y 28865486 PMC5581464

[21] Wang Y Kong H Zeng X Liu W Wang Z Yan X. Activation of NLRP3 inflammasome enhances the proliferation and migration of A549 lung cancer cells. Oncol Rep. (2016) 35:2053–64. 10.3892/or.2016.4569 26782741

[22] Perera AP Sajnani K Dickinson J Eri R Körner H. NLRP3 inflammasome in colitis and colitis-associated colorectal cancer. Mamm Genome. (2018) 29:817–30. 10.1007/s00335-018-9783-2 30206651

[23] Guo W Sun Y Liu W Wu X Guo L Cai P. Small molecule-driven mitophagy-mediated NLRP3 inflammasome inhibition is responsible for the prevention of colitis-associated cancer. Autophagy. (2014) 10:972–85. 10.4161/auto.28374 24879148 PMC4091180

[24] Wei Q Mu K Li T Zhang Y Yang Z Jia X. Deregulation of the NLRP3 inflammasome in hepatic parenchymal cells during liver cancer progression, Lab Invest. (2014) 94:52–62. 10.1038/labinvest.2013.126 24166187

[25] Allen IC TeKippe EM Woodford RM Uronis JM Holl EK Rogers AB. The NLRP3 inflammasome functions as a negative regulator of tumorigenesis during colitis-associated cancer. J Exp Med. (2010) 207:1045–56. 10.1084/jem.20100050 20385749 PMC2867287

[26] Liou GY Storz P. Reactive oxygen species in cancer. Free Radic Res. (2010) 44:479–96. 10.3109/10715761003667554 20370557 PMC3880197

[27] Nakahira K Haspel JA Rathinam VA Lee SJ Dolinay T Lam HC. Autophagy proteins regulate innate immune responses by inhibiting the release of mitochondrial DNA mediated by the NALP3 inflammasome. Nat Immunol. (2011) 12:222–30. 10.1038/ni.1980 21151103 PMC3079381

[28] Bauernfeind F Bartok E Rieger A Franchi L Núñez G Hornung V Cutting edge: reactive oxygen species inhibitors block priming, but not activation, of the nlrp3 inflammasome. J Immunol. (2011) 187:613–7. 10.4049/jimmunol.1100613 21677136 PMC3131480

[29] Juliana C Fernandes-Alnemri T Kang S Farias A Qin F Alnemri ES. Non-transcriptional priming and deubiquitination regulate NLRP3 inflammasome activation. J Biol Chem. (2012) 287:36617–22. 10.1074/jbc.M112.407130 22948162 PMC3476327

[30] Sud'ina GF Mirzoeva OK Pushkareva MA Korshunova GA Sumbatyan NV Varfolomeev SD. Caffeic acid phenethyl ester as a lipoxygenase inhibitor with antioxidant properties. FEBS Lett. (1993) 329:21–4. 10.1016/0014-5793(93)80184-v 7689063

[31] Velazquez C Navarro M Acosta A Angulo A Dominguez Z Robles R. Antibacterial and free-radical scavenging activities of Sonoran propolis. J Appl Microbiol. (2007) 103:1747–56. 10.1111/j.1365-2672.2007.03409.x 17953585

[32] Armutcu F Akyol S Ustunsoy S Turan FF. Therapeutic potential of caffeic acid phenethyl ester and its anti-inflammatory and immunomodulatory effects (Review). Exp Ther Med. (2015) 9:1582–8. 10.3892/etm.2015.2346 26136862 PMC4471667

[33] Tambuwala MM Kesharwani P Shukla R Thompson PD McCarron PA. Caffeic acid phenethyl ester (CAPE) reverses fibrosis caused by chronic colon inflammation in murine model of colitis. Pathol Res Pract. (2018) 214:1909–11. 10.1016/j.prp.2018.08.020 30170869

[34] Tambuwala MM Khan MN Thompson P McCarron PA. Albumin nano-encapsulation of caffeic acid phenethyl ester and piceatannol potentiated its ability to modulate HIF and NF-kB pathways and improves therapeutic outcome in experimental colitis. Drug Deliv Transl Res. (2019) 9:14–24. 10.1007/s13346-018-00597-9 30430451 PMC6328632

[35] Akyol S Ugurcu V Altuntas A Hasgul R Cakmak O Akyol O. Caffeic acid phenethyl ester as a protective agent against nephrotoxicity and/or oxidative kidney damage: a detailed systematic review. Sci World J. (2014) 2014:561971. 10.1155/2014/561971 25003138 PMC4065767

